# Beekeepers Support the Use of RNA Interference (RNAi) to Control *Varroa destructor*

**DOI:** 10.3390/insects15070539

**Published:** 2024-07-18

**Authors:** Rose McGruddy, John Haywood, Philip J. Lester

**Affiliations:** 1 School of Biological Sciences, Victoria University of Wellington, Wellington 6012, New Zealand; phil.lester@vuw.ac.nz; 2 School of Mathematics and Statistics, Victoria University of Wellington, Wellington 6140, New Zealand; john.haywood@vuw.ac.nz

**Keywords:** RNAi, beekeeper opinion, *Varroa destructor*, *Apis mellifera*, honey bee, biotechnology, pest control

## Abstract

**Simple Summary:**

There is a global need for targeted, environmentally friendly, sustainable alternatives to pesticides. In the apicultural industry, the parasitic mite *Varroa destructor* poses a serious threat to honey bee health. Like many other agricultural pests, *Varroa* are primarily managed via pesticides. Whilst these pesticides can effectively control mites, they can also harm bee health, contaminate bee products and pose toxicity risks to beekeepers during application. As a first step to assessing public support for a new biotechnology that could provide a non-toxic alternative to pesticides, beekeepers’ perspectives on a novel *Varroa* control method designed to inhibit protein production, called RNA interference (RNAi), were investigated. The majority of beekeepers surveyed were open to using RNAi treatments against *Varroa*, particularly as it provided a non-toxic alternative to current pesticide options. The major concerns raised were the unknown long-term effects of the RNAi treatment on bee health, potential effects on non-target species that interact with beehives and concern that an uninformed public that might prevent them from accessing a new tool to combat *Varroa*. Surveys such as ours can inform scientists and regulatory authorities on how best to introduce novel biotechnologies for commercial use.

**Abstract:**

Current *Varroa* mite management strategies rely heavily on the use of pesticides, adversely affecting honey bee health and leaving toxic residues in hive products. To explore the likelihood of RNAi technology being utilised as an alternative control method for pests like *Varroa*, the opinions of beekeepers on the use of this new biotechnology were obtained using a mixed-methodology approach. In-person surveys and focus groups using the Q method were conducted to discover the willingness of beekeepers to utilise *Varroa*-targeting RNAi treatments in their hives, and to gain feedback to inform decisions before the implementation of this new technology. Overall, the beekeepers saw potential in RNAi being used to control *Varroa* in their hives and were eager to have access to an alternative to pesticide treatments. Participants raised concerns about unknown long-term effects on bees and other non-target species, and the potential of an uninformed public preventing them from accessing a new *Varroa* treatment. While further research and discussion is needed before RNAi treatments for *Varroa* become commercially available, RNAi technology presents a promising, species-specific and non-toxic solution for *Varroa* management.

## 1. Introduction

European honey bees (*Apis mellifera* Linnaeus) are crucial to global food security and economy due to their pollination services, honey and other valuable hive products [1,2,3]. The parasitic mite *Varroa destructor* (Anderson & Trueman) threatens honey bee health and hive survival on every continent where bees are managed [4,5,6]. These mites latch onto their bee host, pierce through the exoskeleton/cuticle with their mouthparts and feed on the bee, weakening the host’s immune system and shortening its lifespan [7,8,9]. In addition to the direct damage they cause, *Varroa* are vectors of bee viruses [10,11]. High infestations of *Varroa* and the prevalence of their associated viruses can cause honey bee colonies to fail [12,13,14]. Results from annual colony loss surveys across numerous countries have reported *Varroa* to be a primary cause of honey bee colony losses [15,16,17]. These findings suggest that effective *Varroa* control is becoming increasingly difficult for beekeepers to achieve.

Currently, *Varroa* management strategies primarily involve the direct application of chemical treatments within the beehive [18]. These chemical treatments are generally classified as either synthetic or organic pesticides [19]. Synthetic pesticides, by definition, are manmade chemicals that typically work as neurotoxins, which impair or damage the nervous system in invertebrates. By comparison, organic pesticides used in mite control affect other biological systems in a variety of ways depending on the treatment, such as the inhibition of enzymes or minerals important for bodily function [20]. Because these pesticides are derived from or based on natural substances (often sourced from plants), they are referred to as being ‘organic’. This label can be misleading, however, implying that organic *Varroa* treatments are less toxic and safer for bees, when in fact organics can often pose a greater threat to bee health than their synthetic counterparts [21,22]. For organic treatments, the chemicals involved are not species-specific and can harm other animals; they can therefore negatively impact the bees, particularly if the dose is incorrect [23,24,25]. Furthermore, beekeepers must handle organic treatments with care, as they can be corrosive to the eyes and skin [26].

A benefit of organic treatments, however, is that they are naturally occurring chemicals derived from plants and can naturally be present in honey, allowing organic *Varroa* treatments to be applied during the honey flow. As synthetic pesticides are manmade, not naturally derived, regulations are in place worldwide to reduce the exposure of honey to chemicals, and to ensure residues are at safe levels for human consumption, [27,28,29,30]. To maintain chemical residues beneath the required threshold, beekeepers generally avoid applying synthetic treatments during the honey flow. Despite these time constraints, synthetic pesticides are still a popular *Varroa* control method utilised by beekeepers globally [17,19,31]. The frequency and prolonged use of these synthetic pesticides has allowed *Varroa* to develop resistance to many of the available treatments [32,33,34]. The development of resistance to chemical *Varroa* treatments, along with the issue of residues in honey, highlights the need for new modes of action against this honey bee parasite [34,35].

Potential scientific solutions are emerging that could provide new management tools for beekeepers to control *Varroa,* including approaches using biotechnologies like RNA interference (RNAi) [36,37]. RNAi is a natural process in the cells of many organisms that has a role in regulating protein production. Genes contain the code for protein production, with copies of this code in the form of messenger RNA (mRNA), instructing the cell to make a particular protein. The RNAi process is triggered by double-stranded RNA (dsRNA), which causes the targeted mRNAs to be degraded before they can instruct the cell to make a particular protein, thereby temporarily inhibiting that protein’s production [38]. Scientists have been able to harness this RNAi mechanism, using dsRNA to target protein production in pest species with lethal effect [39,40]. These dsRNA treatments can be applied topically or delivered orally, and as the mRNA code for protein production differs across species, dsRNA designed to target a particular species is unlikely to have an effect on other species in the environment [41,42,43,44]. dsRNA treatments are being developed and utilised as non-toxic, species-specific alternatives to pesticides, as there are already dsRNA treatments for invertebrate pest control available for commercial use, and many more are under development with the intent for future release [45,46,47]. Control treatments using dsRNA are also being developed for *Varroa*, as provision of honey bees with *Varroa*-specific dsRNA has proven effective in controlling mite populations [37,48]. One proposed *Varroa* treatment is formulated in a 60% sucrose-based solution, presented in a sealed pouch with perforated openings. This pouch can be positioned across the frames of brood comb within a beehive, allowing honey bees to consume and transfer the dsRNA and sucrose solution via trophallaxis to developing larvae via the brood food. *Varroa* mites come into contact with the dsRNA in the brood cell when they move into the cell during their parasitic lifecycle [49].

In New Zealand, the apiculture industry is of considerable economic value, with pollination services of managed honey bee colonies valued at approximately NZD 5 billion and estimated honey export revenue of NZD 482 million [50]. New Zealand beekeepers have needed to treat their hives for *Varroa* since the parasite was first detected in the early 2000s [51]. *Varroa* is the leading cause of colony loss in New Zealand, and there is evidence to suggest that like many *Varroa* populations overseas, resistance to chemical treatments is likely developing [15,17]. To date, no RNAi treatments have been implemented in New Zealand for pest control purposes. Prior research has been conducted in New Zealand to better understand the public’s views around the use of biotechnologies like RNAi and gene drives for conservational purposes [52,53,54]. Participants in these studies demonstrated difficulty distinguishing between RNAi and gene drives, which is potentially problematic. Gene drives involve genetic modification (GM), thereby creating genetically modified organisms [55]. As the effects of RNAi are temporary and do not permanently alter or eliminate genes within DNA, it has been confirmed as not GM by the Environmental Protection Authority (EPA) in New Zealand, and therefore would legislatively be possible to implement as a new pest control method [56]. Potential public confusion around what RNAi involves and the legality of its use could be problematic for authorities and companies who hope to make RNA treatments available for commercial use.

Society, as the primary consumer of agricultural products, plays an increasingly crucial role in the implementation and regulation of novel biotechnologies [57]. To evaluate the feasibility of using RNA technology for controlling *Varroa* mites, it is advisable to obtain the opinions of those who stand to benefit most: the beekeepers. In this study, a mixed-methodology approach using surveys and the Q method was conducted to provide insight into beekeepers’ perspectives and perceptions on the use of RNAi for *Varroa* management. As RNAi is a novel mode of action for pest control, this study aimed to better understand the different societal perspectives on this technology’s use, with a focus on *Varroa* in order to give scientists, regulatory authorities, and relevant companies insight into the potential implementation of RNAi for pest control.

## 2. Materials and Methods

### 2.1. Participants

The project was designed to help inform scientists and regulatory authorities of beekeepers’ views on the use of RNAi technology to control *Varroa* in beehives. Beekeepers were specifically selected for this study as they have the experience relevant to the topic of *Varroa* control and would benefit most from new management options. To recruit participants, six beekeeping clubs spanning the country of New Zealand were approached (Figure S1). Targeting beekeeping clubs ensured that survey participants were exclusively beekeepers, as these clubs require members to pay annual membership fees and their meetings are not open to the public. Subsequently, between July and October 2023, a *Varroa* RNAi PowerPoint presentation was delivered at each participating club.

Due to its novelty, beekeepers at the club venues were introduced to the concept of RNAi as a *Varroa* control method via a 20-min PowerPoint presentation. PowerPoint was selected as the RNAi educational medium, as informative documents are often not read by participants prior to completing surveys and visual media have proven more successful for informing lay audiences [54,58]. Presenting the PowerPoint in person also ensured that all participants were exposed to the same minimum level of information. A script of the presentation was transcribed and memorised by the presenter to ensure the same wording was used for each beekeeping club to reduce bias. The presentation gave an overview of RNAi technologies via a three-minute animation (Video S1). Additionally, an example case study of a dsRNA treatment for *Varroa* control was presented to the beekeepers. The treatment described is composed of a sucrose solution containing *Varroa*-specific dsRNA, which is fed to the bees in the hive in order to expose *Varroa* to the treatment. Findings from both laboratory and field experiments conducted in New Zealand were shared, discussing benefits and limitations of the efficacy of this RNAi-based treatment in combating *Varroa.* All attendees at each meeting were then invited to complete a short, anonymous survey immediately following the presentation. The survey responses at each regional club varied from 20 to 37 per club, for a total of 175 survey respondents from all meetings across New Zealand. Beekeepers were asked how many beehives they managed to better understand their priorities regarding *Varroa* control, and to gauge the financial implications of *Varroa* management on their livelihoods. The majority of respondents were non-commercial beekeepers, managing fewer than 50 hives, but there were also beekeepers considered to be semi-commercial (51–350 hives) and commercial (351–3000 hives) (Table S1).

### 2.2. Survey Design and Analysis

The survey contained eight questions, predominantly in the Likert style [59], on the topic of current *Varroa* control methods and RNAi technology as a control for *Varroa* (see ‘Beekeeper Survey’ pages 5 & 6, Supplementary Materials). The Likert-scale questions in the survey were developed based on previous studies in the literature that examined attitudes towards novel biotechnologies like RNAi and genetic modification via gene drives [52,54]. The first questions asked the beekeeper how many hives they managed and what *Varroa* control treatments they had used in the last year. Following these questions, beekeepers were then asked to rank five factors that may be considered when choosing a *Varroa* treatment from one to five, with one being the most important, and five being the least important. The five factors were (1) ‘Effort of application’, (2) ‘Over-winter hive survival’, (3) ‘Cost of the *Varroa* treatment’, (4) ‘Level of toxicity of the treatment to bees and/or people’, (5) ‘Maximising honey yield’.

The survey continued with questions asking beekeepers which of the five available statements best represented how they felt about using RNA technology to control *Varroa*, ranging from believing RNA technology should never be used, to the participant having no major concerns about the use of RNAi to control *Varroa.* This question was followed by statements to which beekeepers could respond using a five-point Likert scale of ‘strongly disagree’ (=1); ‘disagree’ (=2); ‘neutral’ (=3); ‘agree’ (=4) and ‘strongly agree’ (=5). One of the statements was focused on trust in New Zealand’s regulatory authority, the Environmental Protection Authority (EPA), to only implement RNAi control treatments if demonstrated to be safe. There was also a question on whether the participant believed RNAi to be genetic modification (and therefore illegal in New Zealand). It should be noted that the question on whether the respondent thought RNAi is genetic modification was added to the survey following feedback from beekeepers at the first regional beekeepers’ club meeting, and therefore there were only 146 responses to that question instead of 175.

Honey is a major source of profit for New Zealand beekeepers, and residues of current chemical *Varroa* treatments need to be below a certain threshold to be considered safe for human consumption. As the example dsRNA-based *Varroa* treatment presented to beekeepers in the PowerPoint presentation is given to bees via a sucrose solution, it is possible that traces may be detected in honey. As such, beekeepers were asked in the survey if they would consume honey containing *Varroa*-dsRNA residues beneath a threshold deemed to be safe by the New Zealand Food Safety authority. This question was also added following feedback from beekeepers at the first regional club meeting, so had 146 responses instead of 175. The final Likert-scale question in the survey asked beekeepers how much they agreed with the statement that they knew enough about RNAi to make an informed decision. At the end of the survey was a question inviting beekeepers to write any specific feedback they had on the survey or any questions/comments on RNAi they wanted to share with the researcher.

The Likert-scale survey results were analysed and presented using RStudio 4.3.2. [60]. To assess the association between beekeepers’ beliefs on whether RNAi is GM, and their perceived knowledge of RNAi, a Spearman’s rank correlation coefficient was calculated. For modelling purposes, respondents who chose not to answer the questions were removed from the analysis. Although a small number of beekeepers chose not to answer all of the questions in the survey, every respondent answered the question on perceived RNAi knowledge.

### 2.3. Q Method Design and Analysis

In addition to the survey, at four of the club meetings, a request was made for three to four volunteers of the beekeepers present at each club to participate in a focus group designed to gather more in-depth feedback on the use of RNAi technology for pest control. To reduce selection bias, volunteers were not told they would receive monetary compensation in the form of a $50 supermarket voucher until after they had agreed to participate. A total of 13 beekeepers participated in the focus groups. The participants represented a mix of non-commercial, semi-commercial and commercial beekeepers, as the number of hives each participant managed ranged from one to 400. Focus group participants also had a large range of years of experience in beekeeping, spanning from a minimum of two years to ~30 years.

The Q method can be used to reveal social perspectives and opinions that exist on an issue, and how the varied views relate to each other. Q statements should all be associated with the topic of focus and be written in a manner that ensures they could be interpreted in different ways [61] (Table S2). In the current study, focus group participants were given a Q method sorting task [62], with statements associated with RNA technology and *Varroa* control, designed to be arranged onto a grid to represent their level of agreement or disagreement with each statement. In total, 25 statements were chosen, as this number meets the minimum requirement to capture points of view on a given topic, without the number of statements overwhelming participants [52,63]. It is recommended that there be fewer participants than statements in a Q study, with the highest recommended ratio allowed being 2:1 [61]. Although it may seem counter-intuitive to not want to maximise the sample size of a scientific study, the purpose of a Q study is to uncover two to five perspectives that capture the breadth of opinions in a group. As such, only 13 participants were included in this Q study, as recommended [63].

Participants were offered a 25-statement sorting grid in the shape of an upside-down pyramid, ranging from −4 (least agree) on one side to +4 (most agree) at the opposite end (Figure S2). The Q method forces a normal distribution, which presumes that every participant agrees and disagrees with the same number of statements, whilst also assigning ‘neutral’ feelings towards the same number of statements for each participant, which is often not the case. Nonetheless, this approach is considered appropriate for this particular methodology as the statements that participants view as the least important are placed in the middle.

The pyramids took approximately 10 to 15 min for participants to complete (Figure S3). Responses to the Q-sort exercise were photographed for analysis, followed by a group discussion on the statements, where participants were invited to discuss and explain their rankings and Q-sorts to the group. Where needed, prompts and questions were provided by the interviewer.

The analysis for the 13 Q-sorts was performed using the package ‘qmethod’ in RStudio [64], with a principal components analysis coupled with a varimax rotation. Factors were produced using this combination as it is considered to be an objective way to extract data and minimises human interference [65]. With the analysis set to produce three different factors, all participants were significantly loaded onto a factor, with acceptable eigenvalues that exceeded the required threshold of one. The factor loading values at minimum needed to exceed 0.5 for each participant and differ by at least 0.14 from the nearest factor loading [65]. In Q methodology, factors with eigenvalues greater than one indicate that the factor explains more variance in the data than a single participant [66]. The three factors were also maintained, as they aligned with perspectives voiced in the focus groups.

Ethics approval for both the anonymous survey and focus groups was obtained from Victoria University of Wellington’s Human Ethics Committee (HEC#31104). The focus groups were semi-structured and varied in length from 40 min to one hour. Focus groups were audio-recorded, and a written-up summary of the main topics discussed in each focus group was transcribed, including quotes of topical interest made by participants. To maintain the anonymity of focus group participants, each individual was assigned a code in place of their name.

## 3. Results

### 3.1. Survey

**Figure 1:**
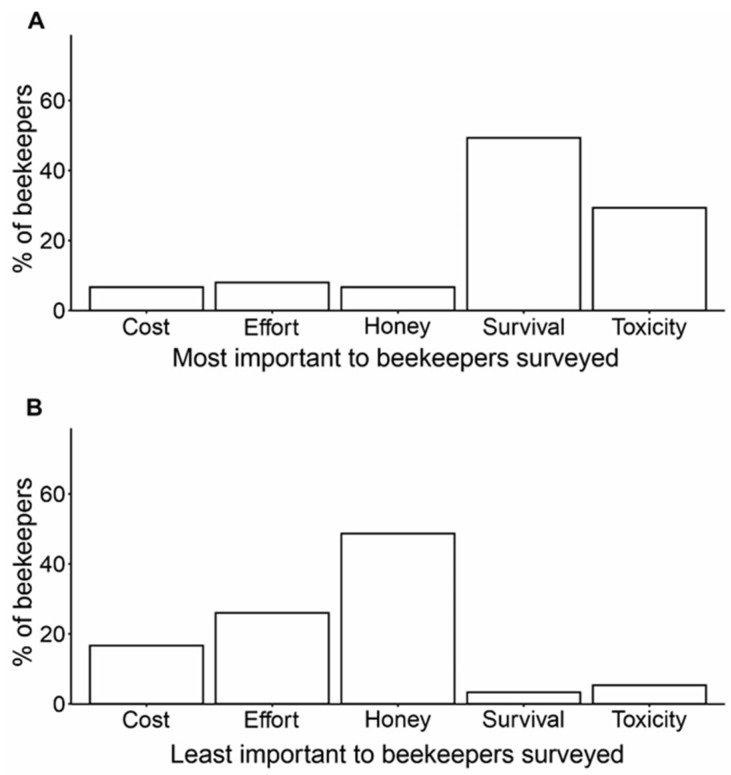
(**A**) The most important factor that is considered when choosing a *Varroa* treatment, according to beekeepers surveyed. The factor options were ‘Cost of the *Varroa* treatment’, ‘Effort of application’, ‘Maximising the amount of honey produced in a season’, ‘Over-winter hive survival’ and ‘Level of toxicity of the treatment to bees and/or people’. The least important factors when choosing a *Varroa* treatment are shown in (**B**). Beekeepers’ responses are presented as a proportion of the 150 beekeepers who answered the question correctly out of the 175 who participated in the survey, as 25 beekeepers chose not to respond or responded with an incorrect ranking scale.

The most important and least important factors when choosing a *Varroa* treatment, according to beekeepers, can be seen in Figure 1. Over-winter hive survival emerged as the most important factor when considering a *Varroa* treatment, with 49.3% of beekeepers ranking hive survival as most important (median = 2, Figure 1A). The second most important factor was toxicity of the treatment, with 29.3% of beekeepers ranking it as first priority and an overall median score of 2. Maximising honey yield was considered the least important factor when considering a *Varroa* treatment, as 48.7% ranked it last (Figure 1B), with an overall median score of 4. The second least important factor to beekeepers, with 26.0% ranking it last, was effort of treatment application (median = 4). Of the 13 commercial and semi-commercial beekeepers, only two ranked maximising honey yield as their top priority when choosing a *Varroa* treatment and five ranked honey yield last. The mean, median and standard deviation (SD) for each factor is presented in Table S3.

**Figure 2:**
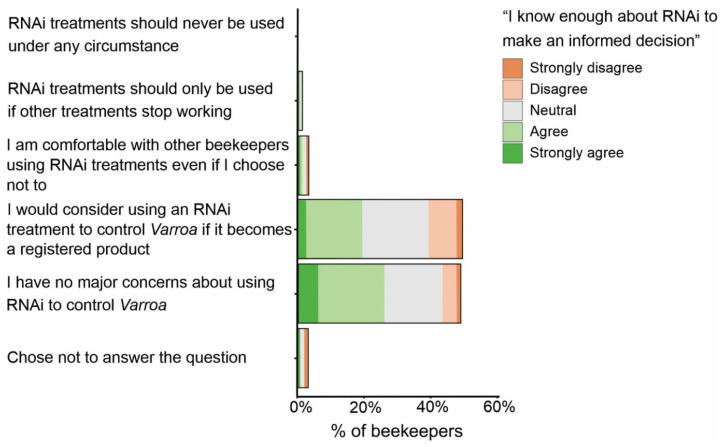
Beekeepers’ responses to a survey on the use of RNAi treatments to control *Varroa* shown as a proportion. Within each grouping of responses is the level of confidence beekeepers had in their own knowledge of RNAi. There were 175 potential respondents for this survey question.

The vast majority of beekeepers surveyed were supportive of RNAi being used as a *Varroa* control, with 46.9% indicating that they would consider using RNAi to control *Varroa* if it became a registered product and a further 46.2% agreeing with the statement that they had no major concerns about RNAi being used to control *Varroa* (Figure 2). Of the beekeepers who demonstrated more reluctance in the use of RNAi, 2.9% were comfortable with other beekeepers using the treatment, even if they chose not to, 1.1% said RNAi treatments should only be used if other treatments stop working, and 2.9% chose not to answer the question. No beekeepers thought RNAi should never be used. It is worth noting that of the beekeepers who said they would consider using RNAi to control *Varroa*, only 39.0% felt they knew enough about RNAi to make an informed decision (agreed or strongly agreed with the statement) (Figure 2). Furthermore, of the beekeepers who would consider using RNAi, a total of 20.8% either disagreed or strongly disagreed with the statement that they knew enough about it. Beekeepers who had no concerns about RNAi being used as a *Varroa* control appeared to be more confident in their perceived knowledge of RNAi, with 53.0% believing they knew enough and only 11.1% believing they did not know enough about RNAi.

**Figure 3:**
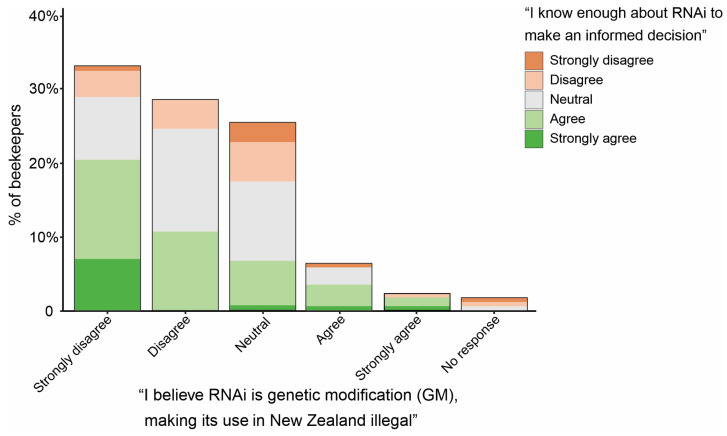
Beekeepers’ responses to a survey question asking if they believed RNAi was genetic modification (GM), making its use in New Zealand illegal. Within each response grouping is the level of confidence beekeepers had in their own knowledge of RNAi. There were 146 potential respondents for this survey question.

With regards to the question as to whether beekeepers believed RNAi is genetic modification, 32.2% strongly disagreed with the statement that they believed RNAi is GM, and a further 29.5% disagreed with the statement (Figure 3). Of the rest of the beekeepers, a notable proportion seemed unsure, as they chose the neutral response (26.0%), and 2.1% chose not to answer the question at all. A combined 10.3% agreed or strongly agreed with the statement that RNAi is GM, thereby making its use in New Zealand illegal under current laws and legislation. The analysis on the relationship between the question on whether RNAi is GM, and the question regarding the beekeeper’s perceived knowledge of RNAi being sufficient found a statistically significant, negative correlation (*r_s_* = −0.21, *P* = 0.010). Beekeepers with higher levels of perceived knowledge of RNAi were less likely to believe RNAi is GM.

**Figure 4:**
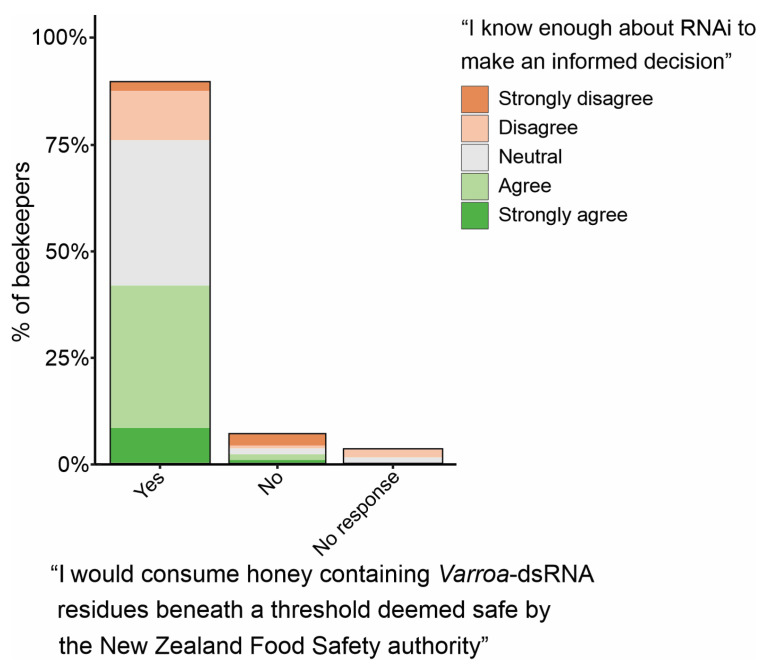
Beekeepers’ feelings on consuming honey containing *Varroa*-dsRNA residues. Within each response grouping is the level of confidence beekeepers had in their own knowledge of RNAi. There were 146 potential respondents for this survey question.

As the dsRNA-based *Varroa* treatment presented to beekeepers prior to the survey is provided to bees via a sucrose solution, beekeepers were asked whether they would consume honey containing *Varroa*-dsRNA residues if it was deemed safe by the New Zealand Food Safety (NZFS) authority. The majority of beekeepers, at 89.7%, did not express concern about ingesting dsRNA residues, whilst 6.8% indicated reservations, and 3.4% were unsure (Figure 4). Among the beekeepers who would consume honey containing dsRNA residues, 46.6% felt they knew enough about RNAi to make an informed decision. A further 38.2% were not sure whether they knew enough, and 15.3% felt they needed to know more about RNAi. Of the beekeepers who said they would not consume honey containing *Varroa*-dsRNA residues, 50.0% felt they did not know enough about RNAi to make an informed decision. This seemingly large proportion should be interpreted with a level of caution, however, as there are fewer beekeepers in this category (*n* = 10) than in the ‘yes’ category (*n* = 131).

It is a requirement in many countries that pesticide products must be approved by that country’s environmental protection authority prior to release. Therefore, as RNA technology cannot be made available as a pest control treatment in New Zealand unless it is approved by the EPA, beekeepers were asked how much trust they had in the EPA to only implement RNAi treatments in beehives if demonstrated to be safe. Most beekeepers appeared to trust the EPA, with 45.7% agreeing with the statement that they trust the EPA, and a further 27.4% strongly agreeing (Figure S4). Not unlike the responses to some of the other survey questions, there was a noteworthy proportion of people who had no strong feelings either way (19.4%), and a small proportion that demonstrated a lack of confidence in the EPA, with 3.4% responding that they disagreed, and another 3.4% that strongly disagreed.

Beekeepers were invited to write any comments or questions they had on RNAi for *Varroa* control at the bottom of the survey. A selection of positive and negative comments is presented in Table S4. The majority of comments from beekeepers demonstrated a willingness to implement RNA technology as a *Varroa* control, with some comments that were very enthusiastic, including enquiries into when the treatment would become available. There were, however, a number of comments that were more cautious about the use of dsRNA treatments for *Varroa*, asking for more information on how RNAi works and calls for further testing of the treatments’ efficacy, safety for bees and long-term effects. Additionally, some beekeepers were wary of how the public would respond to this novel technology. As predicted, there were statements that demonstrated an uncertainty around whether RNAi was GM, as well as concerns that the dsRNA treatment could ‘become wild’ and have unexpected, permanent, long-term effects on bees and other non-target organisms. These statements provide valuable insight into the perspectives of individual beekeepers and aspects of the RNA technology about which they are most concerned.

### 3.2. Focus Groups and Q Method

The Q method is a tool used for providing overarching themes and perspectives surrounding a topic, while illuminating the relationships between differing groups of viewpoints. The Q factor analysis found considerable agreement between groups but revealed three distinct factors that grouped participants who had similarly clustered statements and shared views that set them apart.

#### 3.2.1. Consensus Statements from Q Factor Analyses

Participants demonstrated consensus on nine of the 25 statements regarding RNAi and *Varroa* control (Table S5). The participants agreed that RNAi technology could be an effective new solution for *Varroa* control in their hives. They agreed that they would find it problematic if public backlash prevented them from using a promising, new technology to treat *Varroa*. One participant felt that “*The biggest hurdle will be the public opinion around understanding what RNA is and isn’t*” (participant 2A), with another participant adding “*Some of the public will think this is GM*” (1B). It was pointed out that the name of the RNA technology itself (‘RNA interference’) was problematic, “*Who introduced the word ‘interference’? Because from a public perspective it suggests that you are mucking around with something you shouldn’t be mucking around with*” (1C). One of the participants, however, was more hopeful about public acceptance, stating that “*People are more educated on RNA because of COVID (vaccines)*” (3B).

All participants were open to the idea of using RNA technology to control pests, demonstrated by their strong disagreement with the statement that RNAi should never be used as a control method for pest species. There was an interest from the participants in finding new control methods for *Varroa*, even if the mode of control was unfamiliar. Participants strongly disagreed with the statement that their current *Varroa* treatments were effective. As participant 2B described, “*We need to look for some alternatives, and science is probably the way to do it*”. Participants agreed that they knew enough about RNAi to make an informed decision and disagreed with the statement that they trusted their fellow beekeepers more than scientists when it comes to *Varroa* control.

#### 3.2.2. Distinguishing Statements from Q Factor Analyses

Four of the 13 participants loaded onto Factor 1, with the participants found in two of the four focus groups. Factor 1 accounts for 27.9% of the explained variance and has an eigenvalue of 3.6. Factor 1 participants agreed that their consent to use RNA technology to control *Varroa* is consent for RNAi to be used against other pest species as well (Table 1). They also agreed with the statement that they trust the EPA to only implement RNAi technology in beehives if it is demonstrated to be safe. Participant 1C from the Factor 1 group stated “*This (dsRNA) is a treatment that appears to be relatively safe*”. Although it was not a distinguishing statement for Factor 1, it should be noted that this group strongly agreed with the statement that they would be willing to try RNA technology to control *Varroa* in their beehives.

This group differed from the others in their disagreement with the statement that their opinion counts in the decision whether to use RNAi against *Varroa*. “*People who don’t know anything about it (RNAi) … people with a lack of trust in the EPA … believe their opinion is relevant*” (1A). Factor 1 supports the use of RNA technology to control pest species and trusts regulatory authorities and scientists to only implement dsRNA treatments if they are demonstrated to be ethical and safe.

Six of the 13 participants loaded onto Factor 2. These participants were spread across all four of the focus groups. Factor 2 accounts for 26.6% of the explained variance and has an eigenvalue of 3.5. Like participants in the Factor 1 group, the Factor 2 group strongly agreed that they were willing to try RNAi technology to control *Varroa* in their beehives (Table 1). This group also strongly agreed with the statement that RNAi is a more humane way of controlling *Varroa* mites compared to current chemical treatments. “*All these chemicals have negative effects. The organics (pesticides) are pretty nasty*” (4C).

Their comfort in the use of RNAi, however, did not seem to extend beyond its use for *Varroa* control, as this group strongly disagreed with the statement that their consent to use RNAi technology for *Varroa* control was consent for RNAi to be used against other pests as well. The group also disagreed with the statement that RNAi could be a useful tool for the control of pests/diseases that currently lack an effective treatment. Additionally, they agreed with the statement that they had questions and concerns about the long-term effects of RNAi on the environment and other species, highlighted by participant 3B; “*Like anything new, if it’s not fully researched, the long-term effects are of concern … will it be detrimental to the bee? Will it be detrimental to other species that come into the hive?*” This group felt there is more research to be done before dsRNA treatments for *Varroa* are released for use. The comment by 3A “*I was surprised that you were allowed to do your dsRNA test in the community*” (this participant was referring to field trials discussed in the PowerPoint presentation) suggests that these participants would need more assurances that RNAi is safe before its introduction to the environment.

The Factor 2 group had a neutral response to the statement about having trust in scientists to develop ethical RNA-based pest treatments, whereas the other two groups agreed with the statement. Participant 3A from the Factor 2 group voiced their concern, saying “*There are scientists who are dodgy, who don’t follow the rules*”. Members of this group also differed from the other two groups in their neutral position on whether they would consume honey containing dsRNA residues at levels deemed safe by the New Zealand Food Safety authority. Their response to the question of whether their opinion counts in the decision to use RNAi technology against *Varroa* was also neutral. As a whole, the Factor 2 group members were keen to use the dsRNA treatment in their hives but appeared to be more cautious about dsRNA being used as a control method in general and wanted to see further testing. They were less trusting of regulatory authorities and scientists, but they were neutral regarding the statement that their opinion should count in the decision to use dsRNA against *Varroa*.

The final group, loaded onto Factor 3, contained three participants spread across two focus groups. Factor 3 accounts for 19.9% of the explained variance and has an eigenvalue of 2.6. Unlike the other two groups, who strongly agreed with the statement that they would be willing to try RNA as a *Varroa* control in their beehives, participants in Factor 3 were less enthusiastic, as they only slightly agreed (Table 1). This group instead strongly agreed with the statement that current treatments do not control *Varroa* effectively enough. Discussions around their thoughts on the efficacy of current treatments, however, suggested that the participants in this group believed treatment failure was due to beekeepers applying the treatment incorrectly, rather than the treatment itself being ineffective; “*The treatment does (work), but it’s the person using the treatment … they are not using them correctly*” (1B). Participant 2B added to this, commenting that they had seen how other beekeepers misuse Bayvarol (Bayvarol, Bayer New Zealand Ltd.), which is a synthetic, flumethrin-based *Varroa* treatment. The manufacturer’s instructions for Bayvarol recommend using four treatment strips per brood box, with strip removal after an 8-week period. Participant 2B said the practice of beekeepers under-dosing hives to save money and/or not removing old strips was a common problem; “*I’ve come across hives with as little as one Bayvarol strip, and as many as 13*”.

The Factor 3 group slightly disagreed with the statement that they were comfortable with research being done on RNAi as a control for *Varroa*, although they agreed that the government should invest more funding into RNA technology as a *Varroa* control. The group seemed unsure as to whether RNAi involved genetic modification, and disagreed with the statement that they gave consent for RNA technology to be used against pest species besides *Varroa*. Factor 3 participants were also distinguished by their agreement with the statement that their opinion counts in the decision whether to use RNA technology against *Varroa.* These sentiments suggest that participants in this group wanted more *Varroa* control options to be available and wanted the government to help fund *Varroa* treatment research, but they were just unsure whether RNA technology is the appropriate treatment at this stage.

## 4. Discussion

This study investigated beekeepers’ perspectives on the use of a novel method of pest control for *Varroa* in their beehives, utilising RNAi technology. Engaging with the people who will potentially be using this new technology is an important first step to implementing RNA-based control methods for *Varroa*. This research found an open, positive response towards RNAi technology being used as a *Varroa* control. The vast majority of survey participants, at 93.1%, said that they either had no concerns about RNAi being used to control *Varroa*, or would at least consider using it in their beehives. Such a positive response was surprising, as previous research on the public’s attitudes towards novel biotechnologies has found more reluctance [67]. The motivation underpinning this enthusiasm is likely to be the seemingly urgent need of beekeepers to protect their bees from *Varroa.* Indeed, this attitude was evident in some of the comments made by survey participants, whose statements suggested they did not care what the *Varroa* treatment was, as long as it worked. Comments from beekeepers also highlighted a dislike of using pesticides in beehives and the perception by some beekeepers of a developing resistance of *Varroa* to current treatments. The desperation for new *Varroa* management options is perhaps unsurprising, as annual honey bee colony loss surveys have shown *Varroa* to be a primary driver of hive losses globally [17,68,69,70].

There was consensus among focus group participants that current *Varroa* treatments are not effective enough, and RNAi could be a new solution. One group in particular clarified that the chemicals themselves were not necessarily the problem; rather, it was the poor management practices of some beekeepers that caused chemical treatments to be ineffective. The misuse of chemical strip treatments via underdosing of strips, lack of chemical rotation and failure to remove old strips are all practices believed to increase the speed at which *Varroa* develop resistance to treatments. Due to the biology of honey bees, *Varroa* can be spread relatively quickly between hives, particularly from sick, *Varroa*-infested hives to healthy hives due to robbing [71]. In order to gather resources for their own colony, bees frequently rob from other hives, particularly in autumn as they prepare for the winter [72]. This robbing behaviour allows for re-invasion of *Varroa* into hives that have been treated, and more importantly, potential spread of *Varroa* that have developed resistance to chemical treatments due to a lack of pesticide resistance management. A number of participants from the focus groups suggested that a *Varroa* treatment that was harder for beekeepers to mismanage, and more difficult for *Varroa* populations to develop resistance to, was the way forward. The example *Varroa* dsRNA treatment presented in the talk still has potential to be abused by applying an insufficient amount to hives, thereby failing to control mite levels. However, dsRNA resistance developing in *Varroa* populations would likely be less of an issue, because, although *Varroa* could still develop resistance to dsRNA, this would likely require prolonged, intensive exposure to occur [73].

The first priority for beekeepers when considering which *Varroa* treatment to use in their hives was perhaps, unsurprisingly, ensuring over-winter hive survival. The second highest ranked priority was toxicity of the treatment to bees and/or people. As previously discussed, many of the current *Varroa* treatments are pesticides that can harm honey bee health [21]. Comments made by many beekeepers who participated in the survey and focus groups demonstrated their dislike of having to use harmful pesticides in their hives, but they felt there were few effective alternatives to these treatments. In recent decades, public acceptance of pesticides and poisons has waned, prompting a growing interest in species-specific, environmentally friendly, sustainable alternatives [74,75]. The main advantages of using RNAi as a control method is its expected environmental safety, species-specificity and non-toxic nature, making it safe for beekeepers to apply [41,42,76]. Although the RNAi treatment was presented to the beekeepers as a species-specific, non-toxic control method, some participants still voiced concerns over the unknown long-term effects of the *Varroa*-dsRNA on bee health, non-target species and the environment. It is therefore recommended that scientists conduct further testing on this particular dsRNA product to alleviate these concerns before the product is released.

Honey yield was ranked lowest in the priority list, despite New Zealand honey being a major export commodity [50]. However, the lack of prioritisation of honey yield is likely because the majority of beekeepers surveyed were non-commercial hobbyists, whose livelihood is unlikely to depend on honey production. The quantity of honey a hive produces can be an indicator of overall hive health [77], so a *Varroa* treatment that is less harmful to bees, whilst still controlling mites, is likely to result in a greater honey crop. RNAi technology could provide such an option, provided that dsRNA residues in honey were not an issue to consumers. The proportion of beekeepers in the survey willing to consume honey that might contain dsRNA residues was very high, at 89.7%. High levels of acceptance have also been observed by Shew et al. [78], who asked respondents if they would consume food that was produced using RNAi technology. Their study found that 80–82% of respondents from the USA, Canada and Australia said they would consume RNAi-produced food. Residues of dsRNA treatments are considered to be safe for human consumption [79,80,81], but it is expected that some members of the general public would choose not to consume honey from hives that had been treated with dsRNA. If dsRNA residues were a concern, treatment restrictions during the honey flow could be implemented to reduce the residue risk, as is the practice with current synthetic pesticides.

Whilst the legislation in New Zealand differentiates RNAi technology from genetic modification, and ~60% of the beekeepers believed them to be different technologies, the remaining beekeepers we surveyed were not convinced. The struggle for participants to understand how the two biotechnologies differ and what sets RNAi apart from GM is nothing new [53,54]. It should be noted that an argument has been made to change the legislation to include dsRNA as a potential GM, as it was argued that in some situations there was perhaps evidence that dsRNA could change DNA; however, this argument was rejected by the New Zealand EPA [82]. In the current study, to communicate to beekeepers that dsRNA was not GM, it was explicitly stated in the presentation prior to the survey that “we know using dsRNA does not change the DNA or genome of the species involved, and it degrades naturally”. Despite this statement, 10% of beekeepers believed dsRNA was GM, and a further 26% were unsure whether it was or not. Hesitation in this group of beekeepers appeared to be paired with a perceived lack of knowledge on the topic of RNAi. Their reluctance to immediately accept a technology they feel they know little about is unsurprising, as any new technology or product that is perceived as potentially having unknown consequences on natural ecosystems can be received with public resistance [83].

The cohort in this study were arguably better informed on RNAi technology and its application as a pest control than the general public will be. The focus group participants were particularly confident in their knowledge, as they were in consensus that they knew enough about RNAi to make an informed decision. This response highlights a potential bias in the focus group participants selected, as they likely volunteered to participate because they felt they knew enough on the topic. Beekeepers who felt they did not know enough about RNAi likely thought their contributions to focus group discussions would be inadequate, so did not volunteer, and their viewpoints were not captured. However, as such a large proportion of the 175 beekeepers surveyed were open to using RNAi, the sub-sample of 13 participants for the focus groups was likely to only capture the generally RNAi-positive attitudes regardless.

A primary concern of both survey and focus group participants was the reaction of the general public to a dsRNA-based *Varroa* treatment. The focus group participants agreed that they would find it problematic if public backlash prevented them from using a promising, new technology to treat *Varroa*. It was advised by these beekeepers that public education on RNAi and well-thought-out discussion prior to release would help ensure this novel control option was accepted. Participants feared that the general public would likely believe that RNAi and GM were the same thing, which could hinder the registration and availability of this new *Varroa* treatment option. It is possible, however, that the general public is better educated on RNA technology than it was pre-COVID, and that COVID vaccines have made people more willing to accept RNA technology.

## 5. Conclusions

This study has provided insight on the perspectives of beekeepers regarding the use of a novel biotechnology for control of *Varroa*, a parasitic mite threatening honey bee health. Overall, beekeepers were open, and in some cases, eager, to implement RNAi technologies in their beehives to control *Varroa.* However, some participants requested more research be done on the potential long-term effects of dsRNA treatments on bees and further tests to reduce the chances of *Varroa*-dsRNA having unforeseen effects on non-target species and the environment. Conducting such studies and reporting findings of the research on a forum to which the general public has access could help foster a greater acceptance of this new technology. Education around RNAi and its mode of action is recommended to reduce any potential public backlash stemming from beliefs that RNAi involves genetic modification.

## Figures and Tables

**Table 1:** Statements that distinguished each of the three factor groups, with the distinguishing Z scores for each factor underlined and in bold. Z scores of the other groups have been included for comparison. Statements that participants agreed with have positive Z scores above 0.24 and are highlighted in pale green. Participants agreed the strongest with statements shown in a darker green (Z > 1). For statements with negative Z scores less than −0.24, participants disagreed with the statement (pale peach), and they disagreed the strongest with statements in dark peach (Z < −1). Statements with Z scores close to 0 (0.24 to −0.24) are considered neutral (grey).

				Z Scores		
Group	Participants	ID	Distinguishing Statements	F1	F2	F3
**Factor 1 (F1)**	1A, 1C, 2A,	1	My opinion counts in the decision whether to use RNAi against *Varroa*	**−0.87**	0.02	0.95
	2C	15	Instead of looking for new *Varroa* controls, funding should focus on improving current treatments	**−0.11**	−1.52	−0.97
		19	My consent to use RNAi for *Varroa* control is consent for RNAi to be used against other pest species as well	**0.59**	−1.56	−0.73
		21	I trust the EPA (Environmental Protection Authority) to only implement RNAi in beehives if demonstrated to be safe	**1.18**	−0.58	−0.04
**Factor 2 (F2)**	4C, 3D, 3A,	1	My opinion counts in the decision whether to use RNAi against *Varroa*	−0.87	**0.02**	0.95
	1D, 2D, 3B	7	I trust scientists to develop ethical RNAi-based pest treatments	0.86	**0.24**	1.08
		11	RNAi is a more humane way of controlling *Varroa* mites than current chemical treatments	−0.61	**1.29**	−0.52
		17	I have questions and concerns about the long-term effects of RNAi on the environment and other species	−0.56	**0.36**	−0.55
		19	My consent to use RNAi for *Varroa* control is consent for RNAi to be used against other pest species as well	0.59	**−1.56**	−0.73
		23	I would consume honey containing safe levels of *Varroa*-dsRNA residues as set by New Zealand’s Food Safety authority	1.18	**−0.21**	1.73
		24	Other than *Varroa*, RNAi could be a useful tool for the control of pests/diseases that currently lack an effective treatment	0.49	**−0.25**	0.99
**Factor 3 (F3)**	2B, 1B, 3C	1	My opinion counts in the decision whether to use RNAi against *Varroa*	−0.87	0.02	**0.95**
		4	The current treatments available do not control *Varroa* effectively enough	0.53	1.12	**1.86**
		6	I am comfortable with research being done on RNAi as a control for *Varroa*	1.19	1.63	**−0.62**
		10	The government should invest more funding into RNAi as a control for *Varroa*	0.36	0.59	**1.38**
		13	I would be willing to try dsRNA to control *Varroa* mites in my beehives if it becomes available	1.81	2.04	**0.71**
		14	I believe RNAi is genetic modification, making its use in New Zealand illegal	−1.61	−1.42	**−0.35**
		19	My consent to use RNAi for *Varroa* control is consent for RNAi to be used against other pest species as well	0.59	−1.56	**−0.73**
			Strongly agree			
			Agree			
			Neutral			
			Disagree			
			Strongly disagree			

## Data Availability

The data presented in this study are unavailable due to ethical restrictions.

## Supplementary Materials

The following supporting information can be downloaded at: https://www.mdpi.com/article/10.3390/insects15070539/s1, Figure S1: Map of New Zealand with the location of the six beekeeping clubs that participated in the survey, Video S1: RNAi animation on the use of RNAi as a pest control in New Zealand, Table S1: Number of hives owned by New Zealand beekeepers attending their regional beekeepers’ club meeting that participated in the survey, ‘Beekeeper Survey’: pages 5 & 6, Table S2: The 25 statements beekeepers were provided to complete Q pyramid grids, Figure S2: Format of Q pyramid grid, Figure S3: Example of a finished pyramid completed by one of the focus group participants, Table S3: The mean, median and standard deviation of all beekeepers’ rankings of factors they considered when choosing a *Varroa* treatment, Figure S4: Beekeepers’ trust in the EPA to only implement RNAi treatments in beehives if demonstrated to be safe, Table S4: Written feedback from beekeepers who participated in the survey on using RNAi treatments to control *Varroa* mites, Table S5: Consensus statements and the Z scores calculated for the three different factors in the Q method analyses.
