# Supplementary material for: Beekeepers Support the Use of RNA Interference (RNAi) to Control Varroa destructor
Source: Insects. 2024 Jul 18;15(7):539. doi: 10.3390/insects15070539 (PMC11276693; doi:10.3390/insects15070539)
Supplement: Supplementary file 1 [file insects-15-00539-s001.zip › insects-3113177-supplementary.pdf]

Supplementary Materials

Beekeepers support the use of RNA Interference (RNAi) to control *Varroa destructor*

Rose McGruddy [1]*, John Haywood [2] and Philip J. Lester [1]

[1] School of Biological Sciences, Victoria University of Wellington, Wellington, New Zealand
[2] School of Mathematics and Statistics, Victoria University of Wellington, Wellington, New Zealand

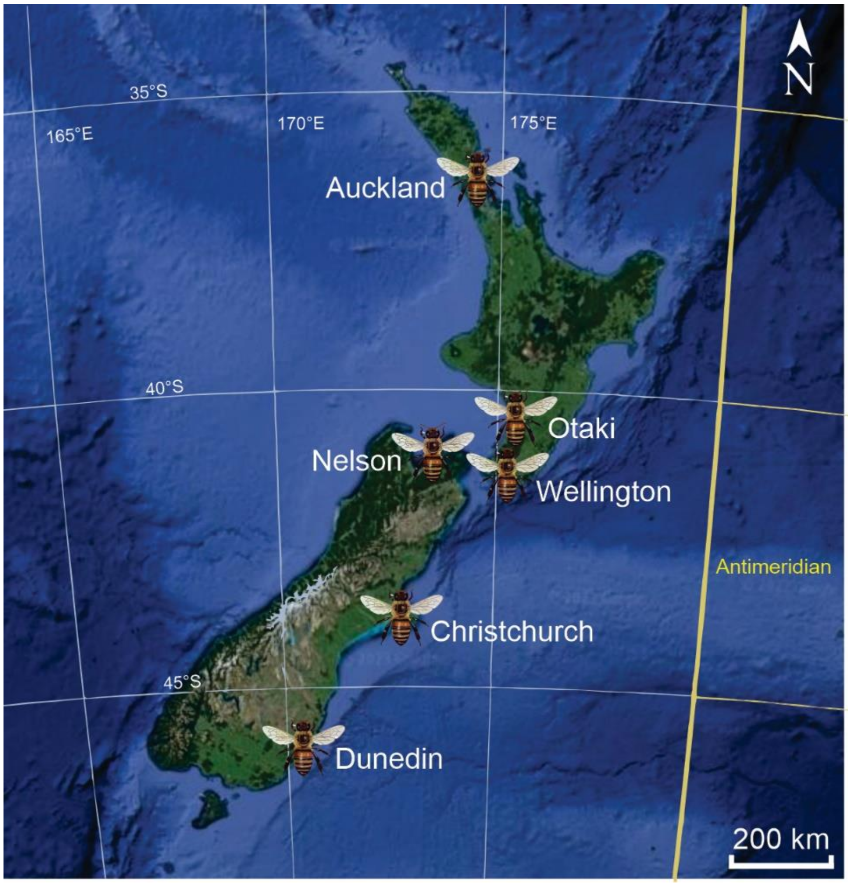

Figure S1. Map of New Zealand (40.9006°S, 174.8860°E), with the location of the six beekeeping clubs that participated in the survey marked with a bee symbol and labelled with the city name. Map sourced from Google Earth (https://earth.google.com).

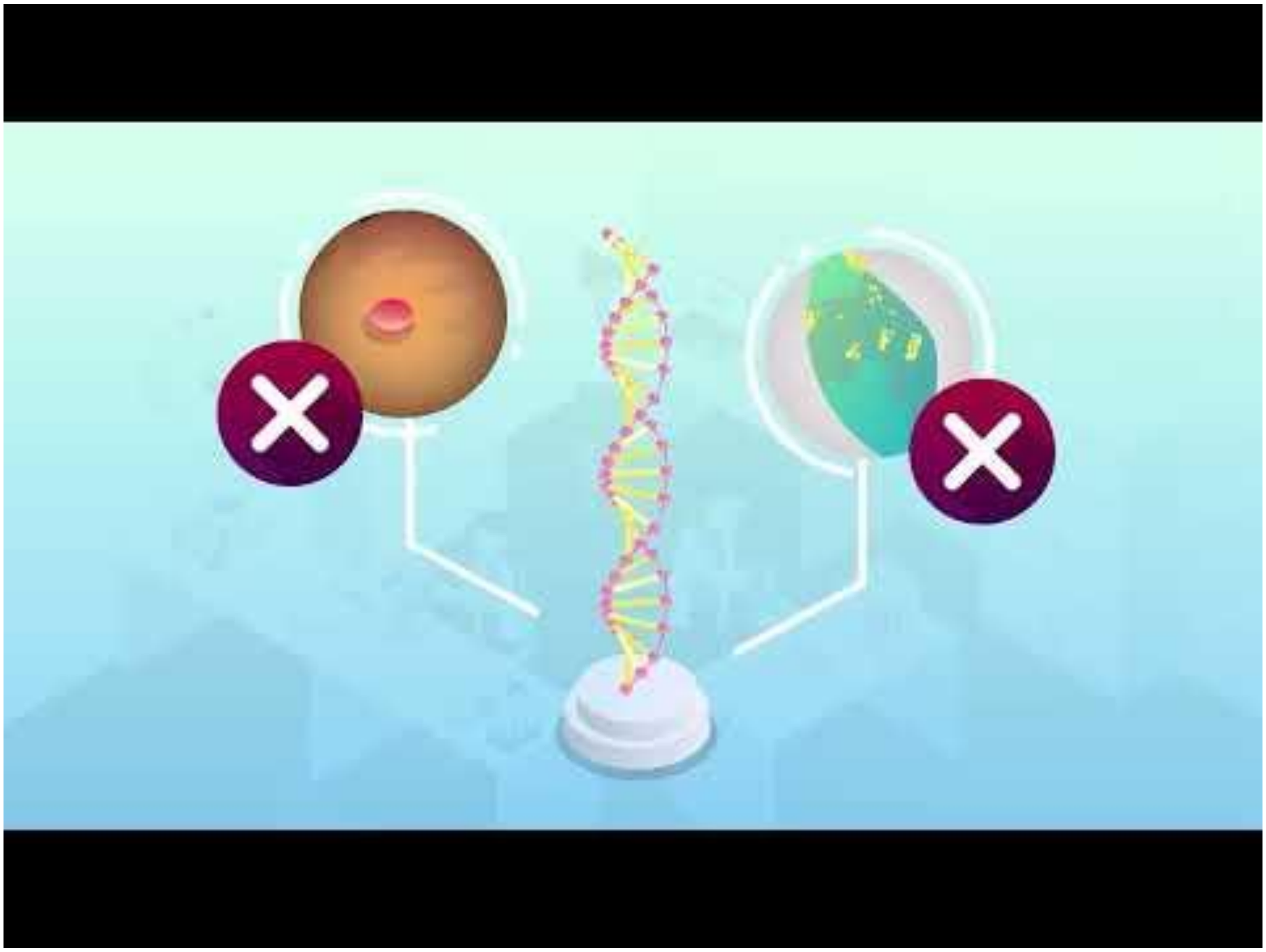

Video S1: RNAi animation on the use of RNAi as a pest control in New Zealand.
https://www.youtube.com/embed/ifKU1zXOrVs?feature=oembed.

Table S1. Number of hives owned by New Zealand beekeepers attending their regional beekeepers' club meeting that participated in the survey. Number of hives has been categorised into non-commercial, semi-commercial and commercial (as outlined by New Zealand's Ministry for Primary Industries, https://www.mpi.govt.nz/dmsdocument/44068-Apiculture-Moniotoring-Report-2020). Beekeepers that had no hives were likely hobbyists that had lost their beehive/s the previous winter, so had no hives at the time of the survey.

	**Non-commercial**		**Semi-commercial**	**Commercial**	
**Number of hives**	**0**	**1-50**	**51-350**	**351-3000**	**3000+**
**Number of beekeepers**	8	154	8	5	0

Beekeeper Survey

☐ I have read the Information Sheet and the project has been explained to me. My questions regarding participation in this survey have been answered to my satisfaction. I understand I can ask further questions at any time. I give consent for my anonymous responses to be used for this study and understand that the findings may be used for a PhD dissertation, academic publications and presentation at conferences.

1. How many beehives do you manage? (Tick one)

☐ 1-50
☐ 51-350
☐ 351-3000
☐ 3000+
☐ I am not currently managing any beehives

2. What *Varroa* treatments did you use over the last year? (Brand or chemical)

3. The table below contains five common priorities that beekeepers may consider when choosing a *Varroa* treatment. Rank the priorities below in order from 1 to 5, with 1 being the most important to you and 5 being the least important.

Rank	
	Effort of application
	Over-winter hive survival
	Cost of the *Varroa* treatment
	Level of toxicity of the treatment to bees and/or people
	Maximising the amount of honey produced in a season

4. Which of these statements best represents how you feel about the use of RNAi as a potential method for controlling *Varroa*? (Tick one)

☐ RNAi treatments should never be used under any circumstance
☐ RNAi treatments should only be used if the other treatments stop working
☐ I am comfortable with other beekeepers using RNAi even if I choose not to
☐ I would consider using RNAi to control *Varroa* if it becomes a registered product
☐ I have no major concerns about using RNAi to control *Varroa*

5. How much do you agree with the following statement: "I trust the Environmental Protection Authority (EPA) to only implement RNAi in beehives if demonstrated to be safe." (Tick one)

☐ Strongly disagree
☐ Disagree
☐ Neutral
☐ Agree
☐ Strongly agree

6. How much do you agree with the following statement “I believe RNAi is genetic modification (GM), making its use in New Zealand illegal.”

☐ Strongly disagree
☐ Disagree
☐ Neutral
☐ Agree
☐ Strongly agree

7. I would consume honey containing *Varroa*-dsRNA residues beneath a threshold deemed safe by the New Zealand Food Safety authority

☐ Yes
☐ No

8. How much do you agree with the following statement: "I know enough about RNAi to make an informed decision." (Tick one)

☐ Strongly disagree
☐ Disagree
☐ Neutral
☐ Agree
☐ Strongly agree

9. Please write any comments on your responses to this survey, or comments/questions you have about the use of RNAi to control *Varroa* below.

Table S2. The 25 statements 13 New Zealand beekeepers were provided to complete Q pyramid grids based on their agreement/disagreement with each statement.

No.	Statement
1	My opinion counts in the decision whether to use RNAi against *Varroa*
2	I am comfortable with other beekeepers using RNAi even if I choose not to
3	RNAi could be an effective new solution for *Varroa* mite control in my beehives
4	The current treatments available do not control *Varroa* effectively enough
5	I know enough about RNAi to make an informed decision
6	I am comfortable with research being done on RNAi as a control for *Varroa*
7	I trust scientists to develop ethical RNAi-based pest treatments
8	*Varroa* mites can be effectively controlled without using RNAi
9	RNAi should never be used as a control method for pest species in New Zealand
10	The government should invest more funding into RNAi as a control for *Varroa*
11	RNAi is a more humane way of controlling *Varroa* mites than current chemical treatments
12	My methods for controlling *Varroa* are effective, and I am not interested in changing them
13	I would be willing to try RNAi to control *Varroa* mites in my beehives if it becomes available
14	I believe RNAi is genetic modification, making its use in New Zealand illegal
15	Instead of looking for new *Varroa* controls, funding should focus on improving current treatments
16	Using RNAi to control pests like *Varroa* would reflect negatively on New Zealand's global image
17	I have questions and concerns about the long-term effects of RNAi on the environment and other species
18	I have concerns regarding the potential impacts of *Varroa*-dsRNA on the health and behaviour of my bees
19	My consent to use RNAi for *Varroa* control is consent for RNAi to be used against other pest species as well
20	I think RNAi needs to be proven effective and safe overseas before it is used as a pest control in New Zealand
21	I trust the EPA (Environmental Protection Authority) to only implement RNAi in beehives if demonstrated to be safe
22	I trust the opinion of my fellow beekeepers more than the opinion of scientists when it comes to *Varroa* control
23	I would consume honey containing safe levels of *Varroa*-dsRNA residues as set by the New Zealand Food Safety authority
24	Other than *Varroa*, RNAi could be a useful tool for the control of pests/diseases that currently lack an effective treatment
25	I would find it problematic if public backlash prevented me from using a promising, new technology to treat *Varroa*

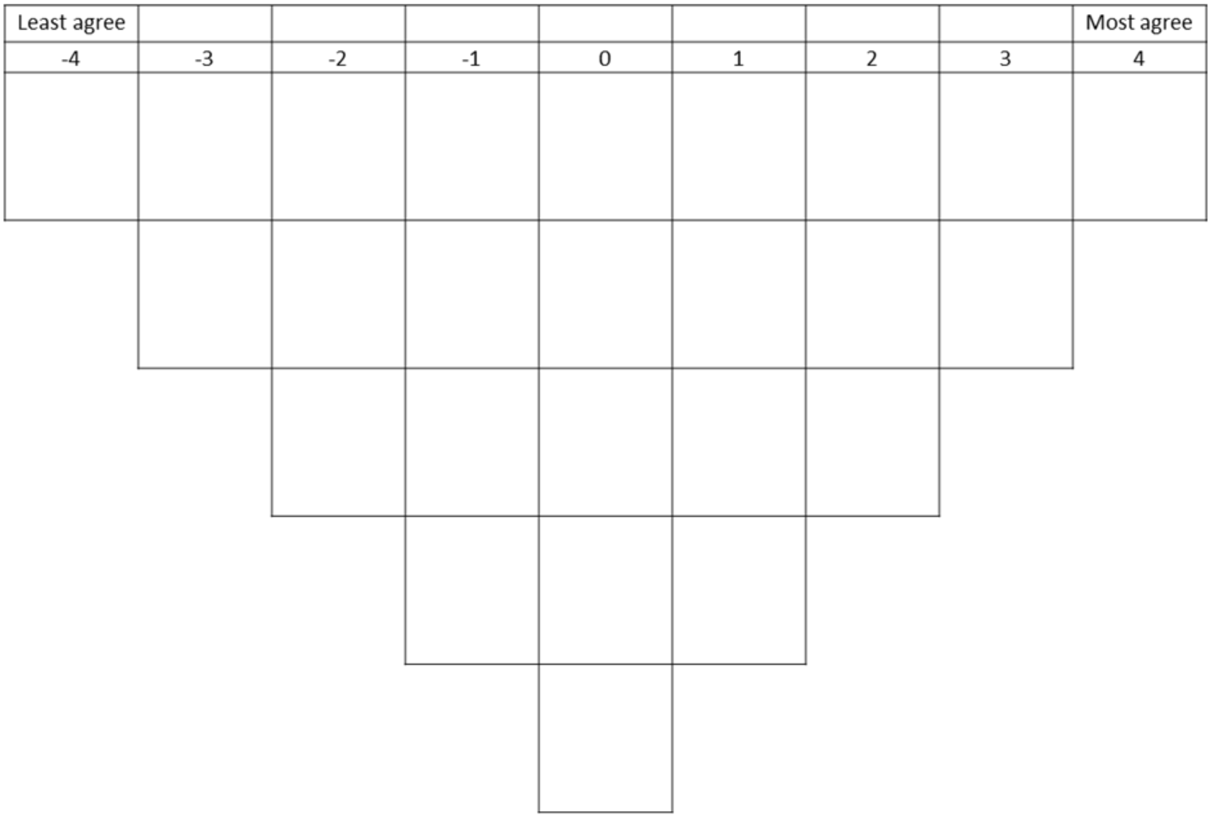

Figure S2. Format of grid where participants were asked to place their 25 statements based on how much they agreed/disagreed with each statement.

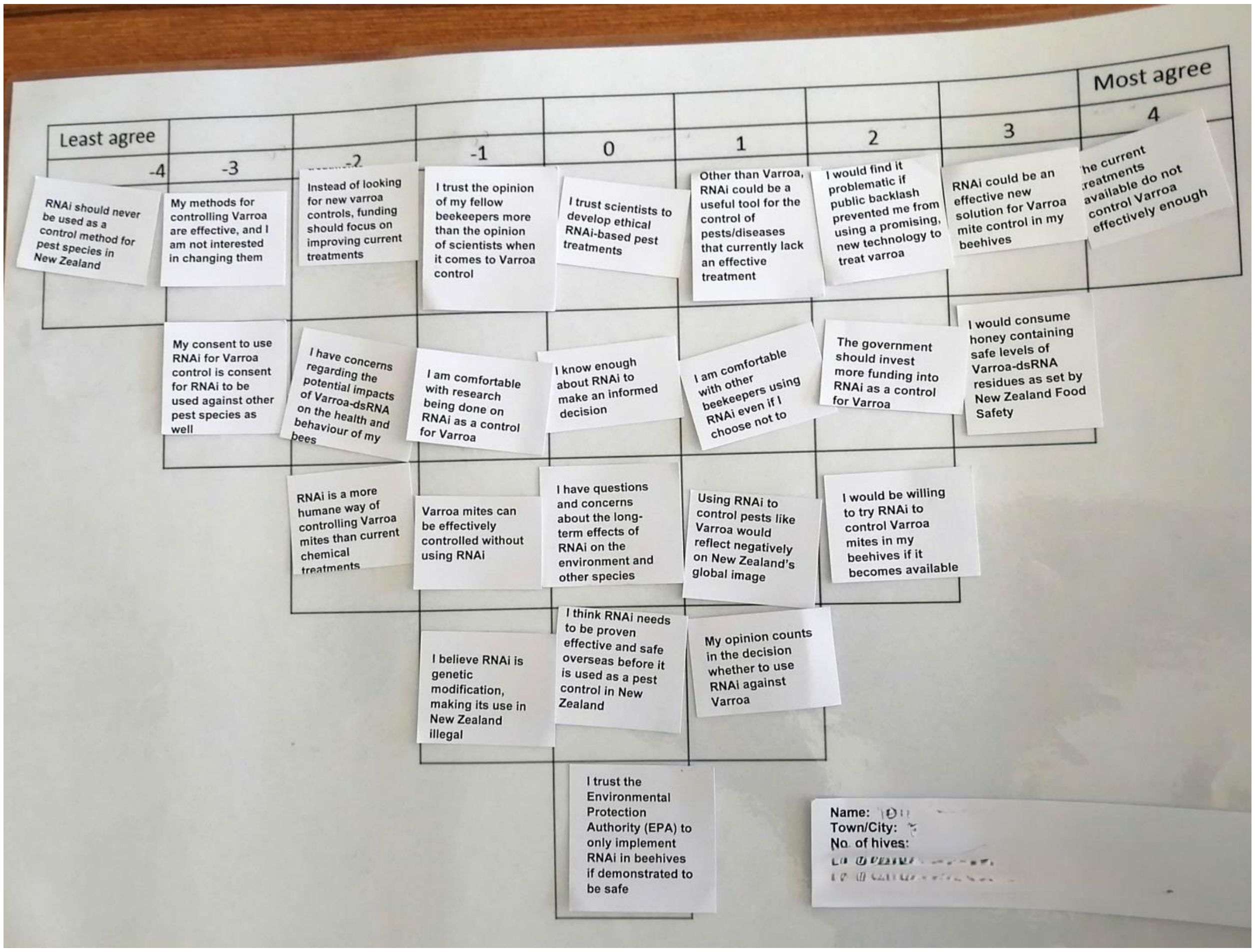

Figure S3. Example of a finished pyramid completed by one of the focus group participants.

Table S3. The mean, median and standard deviation (SD) of all beekeepers' rankings (on a scale of 1-5) of factors they considered when choosing a *Varroa* treatment. The most important factor was ranked with a 1 and the least important factor ranked with a 5. A total of 150 beekeepers answered the question appropriately in the survey.

Factor	Mean	Median	SD
**Effort**	3.51	4	1.23
**Hive survival**	1.88	2	1.10
**Cost**	3.31	3	1.15
**Toxicity**	2.37	2	1.23
**Honey yield**	3.93	4	1.27

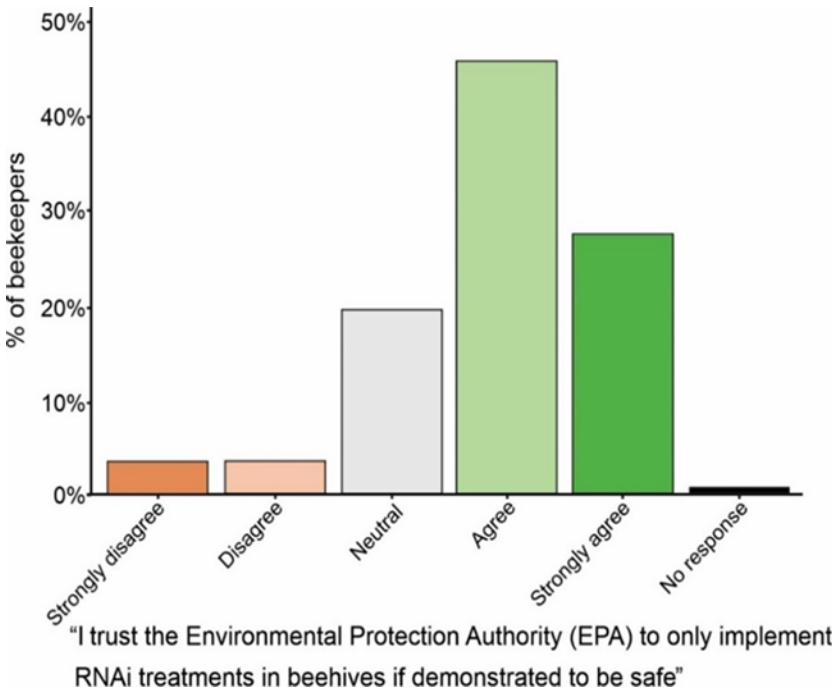

Figure S4. Beekeepers' trust in the EPA to only implement RNAi treatments in beehives if demonstrated to be safe. Responses are presented as a proportion of all 175 beekeepers that participated in the survey.

Table S4. Written feedback from beekeepers that participated in the survey on using RNAi treatments to control *Varroa* mites in their beehives. Comments have been loosely categorised as positive or negative.

**Positive comments**	**Negative comments**
"Unfortunately, the use of chemical strips is becoming useless. So, RNAi would be great."	"I didn't understand what GM was and how introduction of dsRNA was different."
"I am strongly in favour of having this treatment available."	"Would there be a rogue reaction that then goes rampant? How quickly does *Varroa* become immune?"
"A brilliant discovery that I support enthusiastically being developed to combat *Varroa* mites in managed honeybee colonies/hives."	"I need an explanation about the certainty or uncertainty of the RNA modification being incorporated into normal cell processes and this becoming 'wild'."
"Need something better than what we are currently using. Technology like this can be very useful in a number of options/purposes."	"This will be difficult to manage with the public. Marketing of the system will be important in making this palatable to the 'tin hat' types."
"Great to see a very targeted treatment that doesn't damage bees."	"You may get public resistance from fringe 'anti-vax' groups they may associate with mRNA."
"Whatever science can produce that maximises the health and longevity of bees I will support."	"Is it modifying genes directly? Is it simply over-stimulating by increasing the presence of specific RNA?"
"I would love to have a viable method to control *Varroa*, without the use of pesticides."	"What is the long-term accumulation of residues in consumables and what would be the effects long term."

Table S5. Consensus statements and the Z scores calculated for the three different factors that participants were loaded onto. Statements that participants agreed with have positive Z scores above 0.24. Participants agreed most with statements where Z > 1. For statements with negative Z scores less than -0.24 participants disagreed with the statement, and they disagreed most with statements where Z < -1. Statements with Z scores close to 0 (0.24 to -0.24) are considered neutral.

ID	Consensus Statements	Z scores Factor 1	Factor 2	Factor 3
2	I am comfortable with other beekeepers using RNAi as a control even if I choose not to	0.63	0.44	0.21
3	RNAi could be an effective new solution for *Varroa* control in my beehives	1.10	0.81	0.69
5	I know enough about RNAi to make an informed decision	0.37	0.73	0.45
9	RNAi should never be used as a control method for pest species in New Zealand	-1.95	-1.42	-1.94
12	My methods for controlling *Varroa* are effective, and I am not interested in changing them	-1.49	-0.89	-1.47
16	Using RNAi to control pests like *Varroa* would reflect negatively on New Zealand's global image	-0.32	-0.66	-0.50
20	I think RNAi needs to be proven effective and safe overseas before it is used as a pest control in New Zealand	-0.49	-0.68	-0.49
22	I trust the opinion of my fellow beekeepers more than the opinion of scientists when it comes to *Varroa* control	-1.33	-0.84	-0.99
25	I would find it problematic if public backlash prevented me from using a promising, new technology to treat *Varroa*	0.29	0.85	0.56

References
